# Functional evaluation of NK_1_ antagonism on cue reactivity in opiate dependence; An fMRI study

**DOI:** 10.1016/j.drugalcdep.2021.108564

**Published:** 2021-04-01

**Authors:** Leon Fonville, Louise Paterson, Katherine Herlinger, Alexandra Hayes, Raymond Hill, David Nutt, Anne Lingford-Hughes

**Affiliations:** a Division of Psychiatry, Department of Brain Sciences, Imperial College London, United Kingdom; b Department of Metabolism, Digestion and Reproduction, Imperial College London, United Kingdom

**Keywords:** fMRI, Neurokinin, Opiate dependence, Cue reactivity, Caudate, Amygdala

## Abstract

**Background:**

Opiate addiction is a major health challenge with substantial societal cost. Though harm minimisation strategies have been effective, there is a growing need for new treatments for detoxification and relapse prevention. Preclinical research has found neurokinin 1 (NK_1_) receptors have prominent effects on opiate reward and reinforcement, and human studies have found NK_1_ antagonism led to reductions in craving and withdrawal. However, its effect on brain mechanisms in opiate addiction has not yet been examined.

**Methods:**

This study aims to assess the impact of NK_1_ antagonist aprepitant on heroin cue-elicited changes in blood-oxygenation level dependent (BOLD) signal in opiate dependent individuals undergoing detoxification. Participants will attend two scanning sessions and receive a single dose of aprepitant (320 mg) and a placebo in a randomised, cross-over design. During functional magnetic resonance imaging participants will undergo two runs of a cue reactivity task, which consists of passive viewing of drug cues or neutral cues in a block design fashion. We hypothesise that NK_1_ antagonism will attenuate the BOLD response to drug cues in the caudate nucleus and amygdala. Regions of interest were selected based on NK_1_ receptor density and their role in cue reactivity and craving.

## 1 Introduction

Opioid users are at an elevated risk of premature mortality, with overdose as the most common specific cause (Degenhardt et al., 2011; Hser et al., 2015; Ma et al., 2019). Opioid substitution treatment (OST) effectively reduces mortality risk and improves physical and mental health but the extent of its efficacy is affected by patient, environmental, and treatment factors. The aim of OST alongside psychosocial treatments is to reduce so-called ‘on-top’ illicit drug use followed ultimately for some by detoxification to become abstinent. However, the number of individuals who go on to achieve abstinence from opioids remains low (Hser et al., 2015; Soyka et al., 2017; Teesson et al., 2015) as evidenced by the growing proportion of older opioid users in treatment (Carew and Comiskey, 2018; Han et al., 2015). The additional complex drug, health, and social needs of this group – and their impact on treatment outcome – have historically received little attention (Carew and Comiskey, 2018; Dürsteler-MacFarland et al., 2011; Fareed et al., 2009; Gaulen et al., 2017; Rosen et al., 2008). In addition, there has been recent evidence suggesting an age-related increase in methadone-specific deaths among those in treatment (Gao et al., 2020; Pierce et al., 2018). It is therefore important that older clients consider abstinence from opioids. However not only do very few individuals in treatment achieve abstinence, the number leaving treatment opiate free in the UK is declining (National Drug Treatment Monitoring System, 2019). Treatment guidelines in the UK have highlighted detoxification rather than substitution (Clinical Guidelines on Drug Misuse and Dependence Update 2017 Independent Expert Working Group, 2017, Clinical Guidelines on Drug Misuse and Dependence Update 2017 Independent Expert Working Group, 2017), but this can be hard to maintain so that abstinence is achieved (Hser et al., 2015; McKeganey et al., 2006; Soyka et al., 2017). Furthermore, there are limited pharmacological tools to support psychosocial treatment in opiate addiction. Lofexidine, the only approved adjunctive treatment for opiate withdrawal (Pergolizzi et al., 2019), has been unavailable in the UK since 2018 when it was approved for use in the US. Naltrexone is the only licensed medication to help with relapse prevention in abstinent individuals but evidence for its effectiveness is low (Jarvis et al., 2018; Minozzi et al., 2011). Therefore, there is a great unmet need for novel treatments in opioid dependence.

Craving is often cited as a significant risk factor for relapse (Childress et al., 1993; Jasinska et al., 2014). Though it is not a unitary phenomenon (Sayette et al., 2000) – and is further confounded by factors such as context, individual differences, and meta-emotions about craving (Sayette, 2016) – exposure to drug-related cues does typically result in individuals expressing ‘craving’. As such, cue-exposure is often used under laboratory conditions to characterize reactivity and craving. Stress exposure has similarly been linked as a potent trigger for craving and relapse (Preston and Epstein, 2011; Sinha, 2001), and exposure to stress and drug cues show overlapping (Bergquist et al., 2010; Fatseas et al., 2015; Sinha et al., 2003, 2000) and distinct additive (Preston et al., 2018; Preston and Epstein, 2011) physiological and psychological effects on craving.

Exposure to salient drug cues has frequently been used alongside functional magnetic resonance imaging (fMRI) to examine the neural correlates of reactivity and craving. Such cue reactivity (CR) tasks commonly engage attention, memory, and reward mechanisms and it has been shown that blood-oxygen-level-dependent (BOLD) signal changes show considerable overlap across cues showing alcohol, drugs of abuse, food, sex, and gambling (Hill-Bowen et al., 2020; Noori et al., 2016). Furthermore, the strength of the CR response has been linked to subjective value and to clinical outcome; self-reported levels of craving show positive correlations with CR primarily in reward and attention networks (Chase et al., 2011; Jasinska et al., 2014; Yalachkov et al., 2012), and differential CR response is predictive of sustained abstinence and relapse (Courtney et al., 2016; Moeller and Paulus, 2018). Though cue reactivity has not been studied as extensively in opiate dependence, fMRI studies have shown greater BOLD signal across a range of cortical and subcortical areas relative to controls (Li et al., 2012; Murphy et al., 2018; Wei et al., 2019), that is consistent with the broader addiction literature (Moningka et al., 2019). In support of the CR BOLD response as a valid target for treatment in opioid use disorder, it is sensitive to pharmacological manipulation, such that aberrant CR neural response can be attenuated by known efficacious treatments that reduce craving. For example, acute administration of daily methadone dose led to reduction in craving and a reduced BOLD response to drug cues in the insula, amygdala, hippocampal complex, and orbitofrontal cortex (Langleben et al., 2008). Injection of extended release naltrexone led to BOLD signal reductions in the amygdala, caudate, precuneus, and precentral gyrus and an increase in the medial frontal gyrus and precuneus accompanied by reductions in craving, although these did not correlate with the magnitude of change in the BOLD response (Langleben et al., 2014). Decreased signal in the amygdala was also observed following buprenorphine administration, along with reductions in the hippocampus, thalamus and ventral tegmental area (Mei et al., 2010). Given this evidence, novel efficacious drugs might similarly be expected to attenuate aberrant brain responses to cues in a challenge paradigm in opioid dependence, and this attenuation may be indicative of therapeutic benefit.

Neurokinins are a class of neuropeptides that have been linked with drug-related behaviours. In particular, substance P and the neurokinin-1 (NK_1_) receptor have been suggested to play a role in the behavioural response to opioids. NK1 receptors are expressed widely throughout the brain, but the highest density is found in the striatum (Hietala et al., 2005; Okumura et al., 2008). Preclinical studies have found that inhibition of NK_1_ receptors – either via genetic deletion or pharmacological antagonism – can blunt the rewarding properties of opioids and decrease stress-induced reinstatement (Schank, 2020). NK_1_ receptors have been found to positively regulate dopamine signalling within the mesolimbic pathway and the release of substance P in limbic structures has been linked to enhanced stress response, highlighting a multifaceted impact on addiction-related behaviour (Commons, 2010; Ebner and Singewald, 2006; Sinha et al., 2011). Imaging studies of healthy volunteers have found changes in BOLD signal following a single dose of aprepitant, an NK_1_ antagonist, in relation to emotion processing and reward. Aprepitant led to increased BOLD signal in the anterior cingulate and amygdala during presentation of happy facial expressions and in the medial orbitofrontal cortex and precuneus in response to positive compared to neutral words on an emotional Stroop task (McCabe et al., 2009). Similarly, aprepitant led to BOLD signal reductions in the nucleus accumbens during gain anticipation on a monetary incentive delay task (Saji et al., 2013).

The therapeutic potential of NK_1_ has mostly been studied in affective disorders, due to its role in regulating the stress response, but clinical trials have shown mixed results (Czéh et al., 2006). Few human studies have investigated its utility in substance dependence. The first was a clinical trial in alcohol dependence which found reduced craving and stress in response to a selective NK_1_ receptor antagonist, LY686017. In a parallel fMRI paradigm, LY686017 was associated with reduced BOLD signal in the insula in response to negative emotional stimuli and greater BOLD signal to positive stimuli in the striatum compared with a placebo group (George et al., 2008). A follow-up study of aprepitant in comorbid alcohol dependence and post-traumatic stress disorder (PTSD) showed no effect on subjective craving (Kwako et al., 2015), but did result in robust enhancement of BOLD response to aversive stimuli in medial prefrontal cortex, an area commonly associated with hypofunction in PTSD and in substance dependence (Goldstein and Volkow, 2011). Two clinical studies of acute aprepitant have been conducted in opiate dependence. Both observed a reduction in craving and withdrawal and were found to be safe in combination (Jones et al., 2013; Walsh et al., 2013). Interestingly, there was an indication of an acute enhancement of participants’ subjective positive rating of co-administered methadone (Jones et al., 2013) and oxycodone (Walsh et al., 2013) which may point to a μ-opioid-NK_1_ receptor interaction that could have clinical utility (Sandweiss et al., 2018).

Taken together, we suggest that the CR task is a well validated tool to investigate the therapeutic potential of novel drugs in opioid use disorder, and that NK_1_ antagonism shows promise in this regard. NK_1_ antagonists are effective in preclinical models of opioid dependence and there are early indications of potential efficacy in substance dependence. They also have a very good track record of safety in human clinical trials and are available as licensed products.

This research proposal forms part of a larger study called NCORE (Neural Correlates Of Reward and Emotion in opioid dependence), a multi-modal imaging platform that will investigate other well-validated fMRI tasks (https://gtr.ukri.org/projects?ref=MR%2FR024197%2F1). A framework for this approach in substance dependence has been successfully implemented previously in the ICCAM (Imperial College Cambridge Manchester) study (McGonigle et al., 2017; Paterson et al., 2015), and is consistent with the fast-fail approach now adopted by the National Institute of Mental Health (NIMH) for accelerated delivery of novel treatments for depression and anxiety (Krystal et al., 2020, 2019; Pizzagalli et al., 2020), developed in response to the urgent need for new treatments in mental health disorders.

### 1.1 Aims and hypotheses

The NCORE study aims to improve our understanding of opiate dependence during detoxification and early abstinence by characterizing brain processes related to reward processing, emotional processing, and cue reactivity and to assess the impact of a single dose of NK_1_ antagonist aprepitant on these processes to evaluate its therapeutic potential. Here, we will investigate the effect of aprepitant on cue reactivity in individuals on stable doses of methadone. In particular, we will focus on two bilateral regions of interest (ROI) placed on the head of the caudate nucleus and the amygdala. Our first hypothesis focuses on the main effect of the task; we hypothesise that the magnitude of the BOLD response will be greater in response to drug cue stimuli compared with neutral images and this will be observable in our ROIs in terms of percentage signal change.

The dorsal striatum – the caudate nucleus in particular - is typically engaged in cue reactivity (Noori et al., 2016) and craving has been positively linked to local BOLD signal (Li et al., 2012; Lou et al., 2012; Wang et al., 2014) as well as dopamine levels (Koob and Volkow, 2010; Sinha, 2013). We expect that aprepitant will attenuate reactivity to drug cue stimuli, by modulating mesolimbic dopamine levels (Schank, 2020), and this will be observable as a reduction in BOLD response. We will explicitly test this effect in the head of the caudate nucleus as it contains a high density of NK_1_ receptors (Hietala et al., 2005; Okumura et al., 2008). We hypothesise that the magnitude of the BOLD response to drug cue stimuli will be reduced following a single dose of aprepitant compared with a placebo.

The amygdala has been shown to play a mediatory role in the effects of stress and drug cues on craving and drug seeking behaviour (Koob, 2009), and is typically engaged in cue reactivity (Noori et al., 2016). Substance P circuitry is found within the amygdala and is involved in modulating response to stress and reward-related processes (Schank et al., 2012). Previous pharmacological challenges – using naltrexone and buprenorphine – have shown that reductions in craving coincide with reductions in BOLD signal in the amygdala (Langleben et al., 2014; Mei et al., 2010). Therefore, we hypothesise that a single dose of aprepitant will result in a reduction in amygdalar BOLD response to drug cue stimuli when compared with a placebo.

We will further explore the main effect of aprepitant on the BOLD response via a whole-brain analysis. Subsequently, we intend to run a psychophysiological interaction (PPI) analysis using the bilateral head of the caudate nucleus as a seed region to explore the impact on cortico-striatal connectivity.

## 2 Materials and methods

### 2.1 NCORE project

**Fig. 1:**
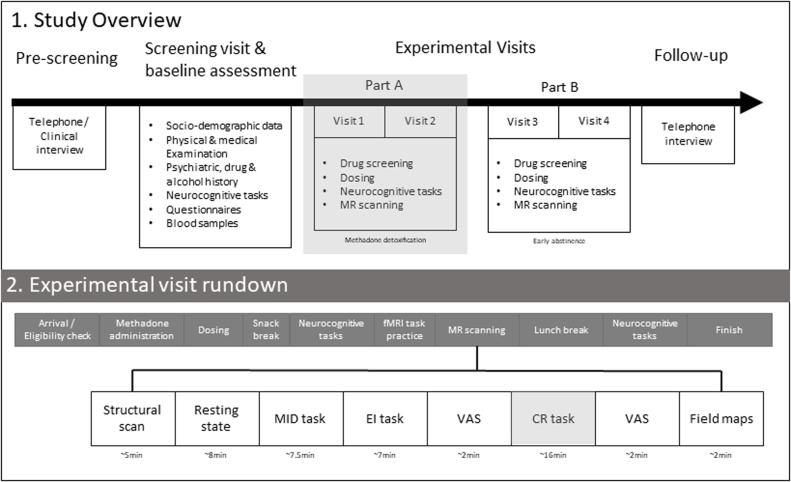
Panel 1 (top) gives an overview of the NCORE project. All participants will have a pre-screening assessment followed by a clinical screening visit and baseline assessment. Participants will receive a single dose of aprepitant or a placebo in a randomised cross-over design in part A and part B. Part A (highlighted in grey) takes place as participants are on a detoxification pathway and part B takes place within four weeks of achieving abstinence. Participants will be followed up for up to 12 months following completion of part B to determine outcomes on substance use and relapse. Panel 2 (bottom) gives a summary of an experimental visit during part A and the order of acquisitions for the imaging protocol. Visual analog scales (VAS) are used to assess anxiety and craving before and after completing two runs of the cue reactivity (CR) task (highlighted in grey).

As previously stated, the NCORE project aims to examine the neural correlates of cue reactivity, monetary reward and negative emotional processing in opiate dependent individuals and the impact of aprepitant. In the wider project, participants will be studied during detoxification from methadone (Part A), and again in early abstinence (Part B), see Fig. 1, across a range of fMRI task paradigms. Ethical approval for this project has been granted by the West London & GTAC ethics committee (REC REF: 19/LO/0971).

This proposal focusses specifically on investigating cue reactivity in Part A, which will be conducted in participants who are on stable doses of methadone. Participants will receive a single, oral dose of placebo and aprepitant (320 mg), in a double-blind, randomised, within-subjects cross-over design.

Participants will be given 320 mg of aprepitant or a placebo in the morning 3−4 hours before MR scanning. Participants will bring their usual methadone dose on the day and consume it under observation. The gap between methadone and aprepitant administration will be kept short. The dose and timing of aprepitant are based on PET occupancy and pharmacokinetic data (Bergström et al., 2004; Majumdar et al., 2006) and this dose will lead to an estimated plasma concentration above 100 ng/mL and achieve 100% NK_1_ receptor occupancy within the striatum. Experimental visits will occur at least 5 days apart; aprepitant has a half-life of 9–13 hours and according to the pharmacokinetic principle it should take 4–5 half-lives to ensure most of the absorbed drug has been eliminated from the body (Ibrahim and Preuss, 2020).

Prior to randomisation, participants will have completed a pre-screening assessment to check eligibility and attended a baseline visit which includes obtaining a comprehensive drug and alcohol history, blood samples for genotyping, completion of neurocognitive tasks, and questionnaires assessing mood and personality traits.

At each experimental visit, eligibility will be checked upon arrival including drug urine screen, alcohol breath, and pregnancy tests. Participants will take their usual daily dose of methadone, which will be the same dose at both visits. The study drug will then be administered. Vital signs – blood pressure, pulse, oxygen saturation, and subjective measures – will be monitored before and after study drug administration as well as at regular intervals throughout the day. If any participant experiences adverse events to aprepitant, these will be recorded. Participants will also complete a set of questionnaires and a cognitive test battery consisting of a probabilistic reward-learning task (Pizzagalli et al., 2008), an opiate dot-probe task (Constantinou et al., 2010), and the emotional processing components of the EMOTICOM test battery (Bland et al., 2016). Scanning will take place approximately 4 hours after dosing and consists of structural and functional image acquisition. Three experimental tasks will be carried out in the scanner after structural and resting state image acquisition. A monetary incentive delay (MID) task and evocative images (EI) task, corresponding to those used in ICCAM (McGonigle et al., 2017), followed by two runs of the cue reactivity task. Participants will be given two scheduled breaks throughout the day during which they are offered food and will be allowed to smoke. A summary of an experimental visit is shown in Fig. 1, panel 2.

Sample size for the NCORE project was based on ROI power calculations using the ICCAM dataset (McGonigle et al., 2017; Paterson et al., 2015). The pwr package (Champely, 2020) was used in R (R Core Team, 2018) to perform an a priori power analysis for paired t-tests comparing mean BOLD percentage signal change between placebo and an NK_1_ antagonist. Though ICCAM did not include a CR task, we believe these will provide a reliable estimate of the effect of NK_1_ antagonism on the BOLD response and will be representative of any effect we may see on the CR task. The effect size for bilateral striatal BOLD signal change during the MID task was d_z_ = 0.49. A power analysis for a one-sided paired *t*-test set to ensure at least 80% power with an alpha of 0.05 suggested that 27 pairs of individuals were required to detect a difference. Likewise, we found an effect size of d_z_ = 0.45 for bilateral amygdalar BOLD signal change during the EI task. Due to uncertainty about the direction of the effect we carried out a power analysis for a two-sided paired *t*-test with an alpha of 0.05 and results suggested 42 pairs of individuals were required to detect a difference with 80% probability. Further details are provided in the supplementary materials and data for the power calculations are plotted in supplementary Fig. 1. We have set a target of 35 individuals scanned twice at part B and will allow for a drop-out from detoxification pathways of up to 50% by setting our scanning target for part A at 70 opiate dependent individuals. We are confident we are sufficiently powered to detect an effect of aprepitant in part A (see supplementary figure 2).

We are adopting the ENIGMA Addiction Cue Reactivity (ACRI) Checklist (Ekhtiari et al., 2020) in our reporting and have added the checklist as supplementary material to highlight where items are addressed in the main body of text.

### 2.2 Participants

Opiate dependent participants will be recruited from substance misuse services within London and surrounding areas. Individuals must have a DSM-5 diagnosis of current severe opioid use disorder, currently be receiving treatment with methadone substitution therapy, and be able to maintain the same stable dose for each experimental visit. Exclusion criteria include any use of relapse-prevention medication, regular on-top use of heroin or other illicit substances, a current substance dependence disorder for any other substance (excluding nicotine), a history or presence of a neurological diagnosis, current or past history of enduring severe mental illness such as schizophrenia or bipolar affective disorder and contraindications to aprepitant or MRI.

In addition, at each experimental scan visit participants will be excluded in the event of a positive alcohol breath test, pregnancy test or urinary screen for drugs of abuse, with the exception of cannabis given the long half-life of cannabinoid metabolites.

### 2.3 Cue-reactivity task

The task is made up of images showing heroin-related cues (e.g. smoking or injecting heroin) or images of neutral pictures (e.g. blowing bubbles or painting) that were matched for hue, brightness, contrast, complexity, and content (e.g. hands, faces). Heroin-related cues include images showing various stages of drug use, drug intake, instruments of drug use, and preparation of drug. These were selected and modified using feedback from a focus group of opiate dependent individuals attending a local treatment service. The task will be undertaken during fMRI scanning and participants will complete 2 runs. During each run, participants will be asked to passively view images presented on the screen. The task is divided into blocks and during each block, 6 images will be presented for 5 s each with a jittered inter-trial interval (200−500 ms). There are 10 blocks in total, 5 that depict heroin-related cues and the other 5 contain neutral images. To make the task less predictable, one block of each will contain 7 images and another set will contain 5 images. Block order is fixed so that each block of neutral images is followed by a block of drug images with a rest block in between. The rest block consists of a fixation cross and lasts 15 s. Each run begins with a block of neutral images. Images within each block will be pseudo-randomised. Duration of each run is 8 min and 20 s. The second run of the task will contain the same images but the order of the blocks is shuffled – whilst keeping the same sequence of neutral blocks followed by drug blocks – and the order of the images within each block is shuffled in a pseudo-randomised order. On the second visit, a different set of images will be presented.

During each visit, we will acquire measures of craving and anxiety and this includes three assessments during the scanning session using a visual analog scale (VAS) with a rating range from 0−10. The first rating is obtained following the MID task. Subsequent ratings are then acquired preceding and following the CR task. The VAS is shown on the screen and the participant will be asked to rate their level of craving over the intercom system to the experimenter. The participant is reminded of their previous rating before being prompted to answer the question. Craving is assessed through: “On a scale of 0−10: How much are you craving right now? (0 = Not at all, 10 = The most ever)”

### 2.4 MRI acquisition

Scanning will take place at the Clinical Imaging Facility at Imperial College London using a 3 T Siemens Magnetom Verio. Participants will undergo preliminary, structural, and resting state sequences followed by three fMRI tasks: a monetary incentive delay task, an evocative images task, and the cue-reactivity task. Participants will spend roughly 1 hour in the scanner.

Functional images will be acquired using a T2*-weighted gradient echo EPI sequence (TR = 1500 ms, TE = 30 ms, flip angle = 62°, FOV =192 mm, voxel size = 3mm^3^, 54 slices, 322 volumes) with in-plane (GRAPPA) acceleration factor of 2 and a multiband acceleration factor of 2. A T1-weighted MPRAGE sequence (TR = 2300 ms, TE = 2.98 ms, TI = 900 ms, flip angle = 9°, FOV = 256 mm, voxel size = 1mm3) will be used to acquire detailed anatomical images and to facilitate spatial transformation to MNI template for whole-brain analysis.

### 2.5 MRI data preprocessing and analysis

Preprocessing will be carried out using fMRIprep 20.0.1 (Esteban et al., 2019), a preprocessing workflow based on Nipype (Gorgolewski et al., 2011), through a singularity container on a high performance cluster. A full boilerplate associated with fMRIprep is provided in the supplementary materials and it provides extensive details on the preprocessing steps. In short, functional data will be slice time corrected and motion corrected before co-registering a reference image obtained from the functional data to the corresponding structural T1-weighted image. The structural image will be warped to MNI space using a non-linear transformation. Transformation parameters for motion correction, coregistration, and warp to MNI space will be concatenated and applied to the functional data in a single step. The functional outputs from fMRIprep in MNI space will be spatially smoothed using a 6 mm full width at half maximum Gaussian kernel.

We will use spherical ROIs with a 5 mm diameter to extract percentage signal change in the head of the caudate nucleus and amygdala. Spheres will be centred around coordinates obtained from a meta-analysis by Noori et al. (2016). Coordinates were global and local maxima located in a cluster of activation across drug cue-reactivity studies that were centred on the head of the caudate nucleus (left: x=-6, y = 10, z = 0; right: x = 8, y = 10, z = 2) and the amygdala (left: x=-22, y=-4, z=-18; right: x = 20, y=-4, z=-12).

First- and second-level data analysis will be carried out using FEAT (Woolrich et al., 2004, 2001), part of FSL (Jenkinson et al., 2012). At the single subject level, each block will be modelled as a boxcar function convolved with the canonical haemodynamic response function. Motion parameters will be included as nuisance regressors alongside confounds obtained from CompCor (Behzadi et al., 2007), as implemented in fMRIprep. Any functional volumes labelled as outliers – a framewise displacement (FD) greater than 0.5 mm or a standardised root mean square of the temporal change (DVARS) greater than 1.5 – will be removed as well. Each run of the task is modelled separately, and a contrast image will be generated for the difference between neutral images and cue images across blocks for each participant. A fixed-effects mid-level analysis will be carried out to obtain an average across runs for each session.

Percentage signal change will be computed from the mid-level contrast of parameter estimate images within ROIs to examine the effect of aprepitant. A paired-samples *t*-test will be used to examine the main effect of aprepitant on BOLD signal changes in the head of the caudate nucleus and the amygdala. A psychophysiological interactions analysis (PPI, O’Reilly et al., 2012) will be carried out by adding the extracted timeseries from the head of the caudate nucleus along with the interaction between the timeseries and the task blocks to a general linear model. The interaction term will be used to identify regions that show task modulated connectivity with the head of the caudate nucleus. This will be done separately for placebo and aprepitant sessions.

At second-level, we will first calculate the difference between the placebo session and the aprepitant session and use these as input for an exploratory whole-brain analysis of the main effect of aprepitant, effectively modelling a one-sample *t*-test. This will be repeated with the outputs from PPI analysis – calculating the difference between whole-brain maps from each PPI analysis and modelling a one-sample *t*-test – to examine differences in connectivity. Statistical significance will be assessed using permutation testing through randomise (Winkler et al., 2014), running 5000 permutations, with threshold-free cluster enhancement to threshold the statistical output (Smith and Nichols, 2009).

The unthresholded statistical outputs from second-level analyses will be uploaded to NeuroVault (https://neurovault.org/) upon completion of the study.

## Role of funding source

This study and the larger NCORE project is funded by the Medical Research Council (MRC), grant number MR/R024197/1.

## Contributors

All authors contributed to the design of the study. LF, LP, and ALH drafted the manuscript with input from KH, AH, RH and DN. All authors approved the final version of the manuscript for submission.

## Declaration of Competing Interest

ALH has received funding for research and/or PhD studentships from Alcarelle Ltd, Lundbeck, GSK; received Honoraria (paid into University account) from Silence Therapeutics, NET Device Corp and consulted by but received no monies from Opiant, Camurus, Dobrin Consulting, Lightlake, GLG; received Honoraria for talks and/or chairing from Janssen-Cilag, Lundbeck, Servier; led the British Association for Psychopharmacology addiction guidelines (2012) that received support from Archimedes Pharma, Lundbeck, Pfizer, Schering. RH is a non-executive director for Asceneuron, Addex, and Avilex. LP has done consultancy work for Alcarelle Ltd. DN has share options in P1Vital and Alcarelle Ltd and is the director of Equasy Enterprises Ltd. He has acted on the advisory boards of Ranvier, Opiant, and COMPASS Pathways, and received additional speaking honoraria from Lundbeck, Janssen, Takeda, and Otsuka. He has acted as an adviser to the British National Formulary.
