# Supplementary material for: Functional evaluation of NK1 antagonism on cue reactivity in opiate dependence; An fMRI study
Source: Drug Alcohol Depend. 2021 Apr 1;221:108564. doi: 10.1016/j.drugalcdep.2021.108564 (PMC8047866; doi:10.1016/j.drugalcdep.2021.108564)
Supplement: Supplementary file 1 [file mmc1.docx]

**Inclusion Criteria**

Participants will be male or female and be over the age of 21. They should be able to read, comprehend and record information written in English and capable of giving written informed consent. Participants need to be healthy as determined by a physician based on a medical evaluation which includes medical history, a physical examination, laboratory tests (if required), and a psychiatric evaluation. All participants will have a DSM-5 diagnosis of current severe opioid use disorder and be treated with methadone substitution therapy. They must be able to maintain the same stable dose across study visits and be committed to a detoxification or tapering pathway.

**Exclusion Criteria**

Participants should not have a current DSM-5 diagnosis of an additional substance dependence disorder except for nicotine. Lifetime history of dependence on other substances will be allowed given very high incidence of co-dependence. Those engaging in regular on-top use of heroin or other opiates or other illicit substances in combination with OST, which in the opinion of the investigators will interfere with subject safety or study integrity, will be excluded. Intoxication at any of the visits, as determined by a breath alcohol test and drug urine screen, will exclude a participant from that study day. A minimum list of drugs that will be screened for include amphetamines, cocaine, opiates, methadone, cannabinoids and benzodiazepines. Positive results for prescribed methadone, buprenorphine, and benzodiazepines will be allowed. Positive results for cannabinoids will be allowed given the long half-life of cannabinoid metabolites. Any participant using relapse-prevention medication – such as disulfiram, acamprosate, naltrexone, baclofen, varenicline, bupropion – within seven days or 5 half-lives (whichever is longer) prior to an experimental visit will also be excluded. Nicotine replacement therapy is allowed.

Current and past medical as well as psychiatric history will be evaluated prior to experimental visits. Participants cannot have active hepatitis or HIV. Screening for Axis I psychiatric diagnoses will be carried out using the Mini-International Neuropsychiatric Interview (MINI) and anyone with a current or past history of enduring severe mental illness, such as schizophrenia, will be ineligible.

Participants should not weigh more than 120kg, have claustrophobia or be unable to lie still on their back inside an MR scanner for up to 90 minutes. Additionally, the presence of a cardiac pacemaker or other electronic device, a history or presence of a neurological diagnosis, clinically significant head injury will also mean they are ineligible to take part.

Contraindications for aprepitant are as follows: hypersensitivity to the active substance or any of the excipients and those taking pimozide, terfenadine, astemizole or cisapride.

Finally, anyone who has recently taken part in a clinical trial and received an investigational medicinal product within 30 days, 5 half-lives, or twice the duration of the biological effect of the investigational product (whichever is longer) prior to the first experimental visit for the NCORE study will be ineligible.

**Power Analysis**

Power calculations for the NCORE study were carried out based on existing data from the Imperial College Cambridge Manchester (ICCAM) platform study. NCORE will use the same tasks as ICCAM to examine reward processing and emotional reactivity; the monetary incentive delay (MID) task and evocative images (EI) task, respectively. We used data from MR scanning sessions where participants were given a placebo (vitamin C) and an NK_1_ antagonist (vofopitant/aprepitant). Only those with a lifetime diagnosis of alcohol dependence (n=20) were selected to assess a relatively homogenous group with substance dependence. Regions of interest (ROI) were made up of 5mm bilateral radius spheres centred on the coordinates specified in McGonigle et al. (2017) – which were based on coordinate-based meta-analyses using activation likelihood estimation – that have shown a robust response on these tasks.

The effect of NK_1_ antagonism on reward processing was examined by extracting mean percentage BOLD signal change from the first-level fixed-effects analysis for the contrast ‘*reward > neutral anticipation’*. Likewise, the effect on emotional reactivity was examined from the first-level contrast *‘aversive > neutral images’*. The same contrasts were used to extract mean percentage BOLD signal change during the placebo session.

Effect size was calculated as Cohen’s d_z_ which uses the standard deviation of the differences between sessions rather than the pooled standard deviation. Outputs are reported in supplementary table 1 and plotted in supplementary figure 1 below.

**Supplementary Table 1.** Summary output of ROI analyses for power calculation and coordinates for each bilateral ROI. Standard deviation is given in parentheses.

|  | Mean Percentage Signal Change | | | Effect Size | MNI Coordinates | | |
| --- | --- | --- | --- | --- | --- | --- | --- |
|  | Placebo | NK_1_ | Difference |  | X | Y | Z |
| Amygdala (EI Task) | 0.07 (0.26) | 0.21 (0.22) | -0.14 (0.28) | 0.45 | ±22 | -4 | -12 |
| Striatum (MID Task) | 0.28 (0.31) | 0.42 (0.39) | -0.14 (0.26) | 0.49 | ±14 | 12 | -4 |


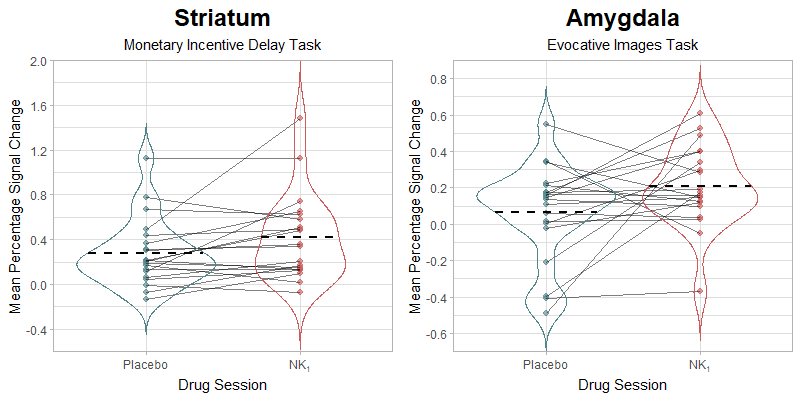


*Supplementary Figure 1.* Violin plot of mean percentage signal change for each session in the relevant ROIs for the MID and EI tasks. Black dotted line represents the group mean.

NCORE sample size was set to detect differences between placebo and an NK_1_ antagonist with 80% probability during part B of the study (see main body of text for description) assuming an attrition rate of up to 50%. Below we demonstrate the estimated statistical power for both part A, where we will recruit 70 participants, and part B given the reported effect sizes from ICCAM. Statistical power for the striatal ROI was based on a one-sided paired t-test whereas for the amygdala ROI this was a two-sided paired t-test.


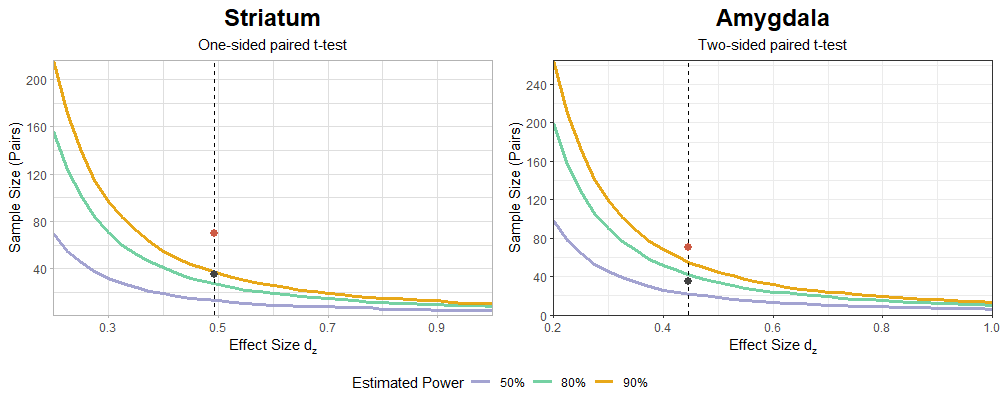


*Supplementary Figure 2.* Required sample size to achieve a minimum statistical power given a certain effect size. The red dot shows the planned total sample size for part A (n=70) and the black dot shows the sample size for part B (n=35).

**fMRIprep boilerplate**

Results included in this manuscript come from preprocessing performed using *fMRIPrep* 20.0.1 (Esteban, Markiewicz, et al. (2018); Esteban, Blair, et al. (2018); RRID:SCR_016216), which is based on *Nipype* 1.4.2 (Gorgolewski et al. (2011); Gorgolewski et al. (2018); RRID:SCR_002502).

*Anatomical data preprocessing*

The T_1_-weighted (T_1_w) image was corrected for intensity non-uniformity (INU) with N4BiasFieldCorrection (Tustison et al. 2010), distributed with ANTs 2.2.0 (Avants et al. 2008, RRID:SCR_004757), and used as T_1_w-reference throughout the workflow. The T_1_w-reference was then skull-stripped with a *Nipype* implementation of the antsBrainExtraction.sh workflow (from ANTs), using OASIS30ANTs as target template. Brain tissue segmentation of cerebrospinal fluid (CSF), white-matter (WM) and gray-matter (GM) was performed on the brain-extracted T_1_w using fast (FSL 5.0.9, RRID:SCR_002823, Zhang, Brady, and Smith 2001). Brain surfaces were reconstructed using recon-all (FreeSurfer 6.0.1, RRID:SCR_001847, Dale, Fischl, and Sereno 1999), and the brain mask estimated previously was refined with a custom variation of the method to reconcile ANTs-derived and FreeSurfer-derived segmentations of the cortical gray-matter of Mindboggle (RRID:SCR_002438, Klein et al. 2017). Volume-based spatial normalization to one standard space (MNI152NLin2009cAsym) was performed through nonlinear registration with antsRegistration (ANTs 2.2.0), using brain-extracted versions of both T_1_w reference and the T_1_w template. The following template was selected for spatial normalization: *ICBM 152 Nonlinear Asymmetrical template version 2009c* [Fonov et al. (2009), RRID:SCR_008796; TemplateFlow ID: MNI152NLin2009cAsym].

*Functional data preprocessing*

For each of the 4 BOLD runs found per subject (across all tasks and sessions), the following preprocessing was performed. First, a reference volume and its skull-stripped version were generated using a custom methodology of *fMRIPrep*. A B0-nonuniformity map (or *fieldmap*) was estimated based on a phase-difference map calculated with a dual-echo GRE (gradient-recall echo) sequence, processed with a custom workflow of *SDCFlows* inspired by the [epidewarp.fsl script](http://www.nmr.mgh.harvard.edu/~greve/fbirn/b0/epidewarp.fsl) and further improvements in HCP Pipelines (Glasser et al. 2013). The *fieldmap* was then co-registered to the target EPI (echo-planar imaging) reference run and converted to a displacements field map (amenable to registration tools such as ANTs) with FSL’s fugue and other *SDCflows* tools. Based on the estimated susceptibility distortion, a corrected EPI (echo-planar imaging) reference was calculated for a more accurate co-registration with the anatomical reference. The BOLD reference was then co-registered to the T_1_w reference using bbregister (FreeSurfer) which implements boundary-based registration (Greve and Fischl 2009). Co-registration was configured with six degrees of freedom. Head-motion parameters with respect to the BOLD reference (transformation matrices, and six corresponding rotation and translation parameters) are estimated before any spatiotemporal filtering using mcflirt (FSL 5.0.9, Jenkinson et al. 2002). BOLD runs were slice-time corrected using 3dTshift from AFNI 20160207 (Cox and Hyde 1997, RRID:SCR_005927). The BOLD time-series (including slice-timing correction when applied) were resampled onto their original, native space by applying a single, composite transform to correct for head-motion and susceptibility distortions. These resampled BOLD time-series will be referred to as *preprocessed BOLD in original space*, or just *preprocessed BOLD*. The BOLD time-series were resampled into standard space, generating a *preprocessed BOLD run in MNI152NLin2009cAsym space*. First, a reference volume and its skull-stripped version were generated using a custom methodology of *fMRIPrep*. Several confounding time-series were calculated based on the *preprocessed BOLD*: framewise displacement (FD), DVARS and three region-wise global signals. FD and DVARS are calculated for each functional run, both using their implementations in *Nipype* (following the definitions by Power et al. 2014). The three global signals are extracted within the CSF, the WM, and the whole-brain masks. Additionally, a set of physiological regressors were extracted to allow for component-based noise correction (*CompCor*, Behzadi et al. 2007). Principal components are estimated after high-pass filtering the *preprocessed BOLD* time-series (using a discrete cosine filter with 128s cut-off) for the two *CompCor* variants: temporal (tCompCor) and anatomical (aCompCor). tCompCor components are then calculated from the top 5% variable voxels within a mask covering the subcortical regions. This subcortical mask is obtained by heavily eroding the brain mask, which ensures it does not include cortical GM regions. For aCompCor, components are calculated within the intersection of the aforementioned mask and the union of CSF and WM masks calculated in T_1_w space, after their projection to the native space of each functional run (using the inverse BOLD-to-T_1_w transformation). Components are also calculated separately within the WM and CSF masks. For each CompCor decomposition, the *k* components with the largest singular values are retained, such that the retained components’ time series are sufficient to explain 50 percent of variance across the nuisance mask (CSF, WM, combined, or temporal). The remaining components are dropped from consideration. The head-motion estimates calculated in the correction step were also placed within the corresponding confounds file. The confound time series derived from head motion estimates and global signals were expanded with the inclusion of temporal derivatives and quadratic terms for each (Satterthwaite et al. 2013). Frames that exceeded a threshold of 0.5 mm FD or 1.5 standardised DVARS were annotated as motion outliers. All resamplings can be performed with *a single interpolation step* by composing all the pertinent transformations (i.e. head-motion transform matrices, susceptibility distortion correction when available, and co-registrations to anatomical and output spaces). Gridded (volumetric) resamplings were performed using antsApplyTransforms (ANTs), configured with Lanczos interpolation to minimize the smoothing effects of other kernels (Lanczos 1964). Non-gridded (surface) resamplings were performed using mri_vol2surf (FreeSurfer).

Many internal operations of *fMRIPrep* use *Nilearn* 0.6.2 (Abraham et al. 2014, RRID:SCR_001362), mostly within the functional processing workflow. For more details of the pipeline, see [the section corresponding to workflows in *fMRIPrep*’s documentation](https://fmriprep.readthedocs.io/en/latest/workflows.html).

*Copyright Waiver*

The above boilerplate text was automatically generated by fMRIPrep with the express intention that users should copy and paste this text into their manuscripts *unchanged*. It is released under the [CC0](https://creativecommons.org/publicdomain/zero/1.0/) license.

**Additional references**

McGonigle, J., Murphy, A., Paterson, L.M., Reed, L.J., Nestor, L., Nash, J., Elliott, R., Ersche, K.D., Flechais, R.S., Newbould, R., Orban, C., Smith, D.G., Taylor, E.M., Waldman, A.D., Robbins, T.W., Deakin, J.W., Nutt, D.J., Lingford-Hughes, A.R., Suckling, J., 2017. The ICCAM platform study: An experimental medicine platform for evaluating new drugs for relapse prevention in addiction. Part B: fMRI description. J Psychopharmacol 31, 3–16. <https://doi.org/10.1177/0269881116668592>

Abraham, Alexandre, Fabian Pedregosa, Michael Eickenberg, Philippe Gervais, Andreas Mueller, Jean Kossaifi, Alexandre Gramfort, Bertrand Thirion, and Gael Varoquaux. 2014. “Machine Learning for Neuroimaging with Scikit-Learn.” *Frontiers in Neuroinformatics* 8. <https://doi.org/10.3389/fninf.2014.00014>.

Avants, B.B., C.L. Epstein, M. Grossman, and J.C. Gee. 2008. “Symmetric Diffeomorphic Image Registration with Cross-Correlation: Evaluating Automated Labeling of Elderly and Neurodegenerative Brain.” *Medical Image Analysis* 12 (1): 26–41. <https://doi.org/10.1016/j.media.2007.06.004>.

Behzadi, Yashar, Khaled Restom, Joy Liau, and Thomas T. Liu. 2007. “A Component Based Noise Correction Method (CompCor) for BOLD and Perfusion Based fMRI.” *NeuroImage* 37 (1): 90–101. <https://doi.org/10.1016/j.neuroimage.2007.04.042>.

Cox, Robert W., and James S. Hyde. 1997. “Software Tools for Analysis and Visualization of fMRI Data.” *NMR in Biomedicine* 10 (4-5): 171–78. [https://doi.org/10.1002/(SICI)1099-1492(199706/08)10:4/5<171::AID-NBM453>3.0.CO;2-L](https://doi.org/10.1002/(SICI)1099-1492(199706/08)10:4/5%3c171::AID-NBM453%3e3.0.CO;2-L).

Dale, Anders M., Bruce Fischl, and Martin I. Sereno. 1999. “Cortical Surface-Based Analysis: I. Segmentation and Surface Reconstruction.” *NeuroImage* 9 (2): 179–94. <https://doi.org/10.1006/nimg.1998.0395>.

Esteban, Oscar, Ross Blair, Christopher J. Markiewicz, Shoshana L. Berleant, Craig Moodie, Feilong Ma, Ayse Ilkay Isik, et al. 2018. “FMRIPrep.” *Software*. Zenodo. <https://doi.org/10.5281/zenodo.852659>.

Esteban, Oscar, Christopher Markiewicz, Ross W Blair, Craig Moodie, Ayse Ilkay Isik, Asier Erramuzpe Aliaga, James Kent, et al. 2018. “fMRIPrep: A Robust Preprocessing Pipeline for Functional MRI.” *Nature Methods*. <https://doi.org/10.1038/s41592-018-0235-4>.

Fonov, VS, AC Evans, RC McKinstry, CR Almli, and DL Collins. 2009. “Unbiased Nonlinear Average Age-Appropriate Brain Templates from Birth to Adulthood.” *NeuroImage* 47, Supplement 1: S102. <https://doi.org/10.1016/S1053-8119(09)70884-5>.

Glasser, Matthew F., Stamatios N. Sotiropoulos, J. Anthony Wilson, Timothy S. Coalson, Bruce Fischl, Jesper L. Andersson, Junqian Xu, et al. 2013. “The Minimal Preprocessing Pipelines for the Human Connectome Project.” *NeuroImage*, Mapping the connectome, 80: 105–24. <https://doi.org/10.1016/j.neuroimage.2013.04.127>.

Gorgolewski, K., C. D. Burns, C. Madison, D. Clark, Y. O. Halchenko, M. L. Waskom, and S. Ghosh. 2011. “Nipype: A Flexible, Lightweight and Extensible Neuroimaging Data Processing Framework in Python.” *Frontiers in Neuroinformatics* 5: 13. <https://doi.org/10.3389/fninf.2011.00013>.

Gorgolewski, Krzysztof J., Oscar Esteban, Christopher J. Markiewicz, Erik Ziegler, David Gage Ellis, Michael Philipp Notter, Dorota Jarecka, et al. 2018. “Nipype.” *Software*. Zenodo. <https://doi.org/10.5281/zenodo.596855>.

Greve, Douglas N, and Bruce Fischl. 2009. “Accurate and Robust Brain Image Alignment Using Boundary-Based Registration.” *NeuroImage* 48 (1): 63–72. <https://doi.org/10.1016/j.neuroimage.2009.06.060>.

Jenkinson, Mark, Peter Bannister, Michael Brady, and Stephen Smith. 2002. “Improved Optimization for the Robust and Accurate Linear Registration and Motion Correction of Brain Images.” *NeuroImage* 17 (2): 825–41. <https://doi.org/10.1006/nimg.2002.1132>.

Klein, Arno, Satrajit S. Ghosh, Forrest S. Bao, Joachim Giard, Yrjö Häme, Eliezer Stavsky, Noah Lee, et al. 2017. “Mindboggling Morphometry of Human Brains.” *PLOS Computational Biology* 13 (2): e1005350. <https://doi.org/10.1371/journal.pcbi.1005350>.

Lanczos, C. 1964. “Evaluation of Noisy Data.” *Journal of the Society for Industrial and Applied Mathematics Series B Numerical Analysis* 1 (1): 76–85. <https://doi.org/10.1137/0701007>.

Power, Jonathan D., Anish Mitra, Timothy O. Laumann, Abraham Z. Snyder, Bradley L. Schlaggar, and Steven E. Petersen. 2014. “Methods to Detect, Characterize, and Remove Motion Artifact in Resting State fMRI.” *NeuroImage* 84 (Supplement C): 320–41. <https://doi.org/10.1016/j.neuroimage.2013.08.048>.

Satterthwaite, Theodore D., Mark A. Elliott, Raphael T. Gerraty, Kosha Ruparel, James Loughead, Monica E. Calkins, Simon B. Eickhoff, et al. 2013. “An improved framework for confound regression and filtering for control of motion artifact in the preprocessing of resting-state functional connectivity data.” *NeuroImage* 64 (1): 240–56. <https://doi.org/10.1016/j.neuroimage.2012.08.052>.

Tustison, N. J., B. B. Avants, P. A. Cook, Y. Zheng, A. Egan, P. A. Yushkevich, and J. C. Gee. 2010. “N4ITK: Improved N3 Bias Correction.” *IEEE Transactions on Medical Imaging* 29 (6): 1310–20. <https://doi.org/10.1109/TMI.2010.2046908>.

Zhang, Y., M. Brady, and S. Smith. 2001. “Segmentation of Brain MR Images Through a Hidden Markov Random Field Model and the Expectation-Maximization Algorithm.” *IEEE Transactions on Medical Imaging* 20 (1): 45–57. <https://doi.org/10.1109/42.906424>.
